# Nail changes in systemic amyloidosis

**DOI:** 10.1002/ccr3.4685

**Published:** 2021-08-21

**Authors:** Noureddine Litaiem, Ines Chabchoub, Soumaya Gara, Maroua Slouma, Mohamed Salah Hamdi, Faten Zeglaoui

**Affiliations:** ^1^ Department of Dermatology Charles Nicolle Hospital Tunis Tunisia; ^2^ Faculty of Medicine of Tunis University of Tunis El Manar Tunis Tunisia; ^3^ Department of Internal Medicine Military Hospital of Instruction Tunis Tunisia; ^4^ Department of Internal Medicine Charles Nicolle Hospital Tunis Tunisia

**Keywords:** amyloidosis, dermoscopy, nails, splinter hemorrhage, trachyonychia

## Abstract

Nail examination must be an essential part of physical examination in our daily clinical practice, as nail changes may be the revealing sign of systemic diseases in the absence of other alarming signs.

## Graphical abstract

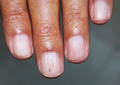

## 1 CASE HISTORY

Nail changes represent a rare sign of systemic amyloidosis. However, recognition of this phenomenon is of key importance for clinicians as it may lead to perform appropriate investigations and diagnose this systemic disease at an early stage.

**FIGURE 1:**
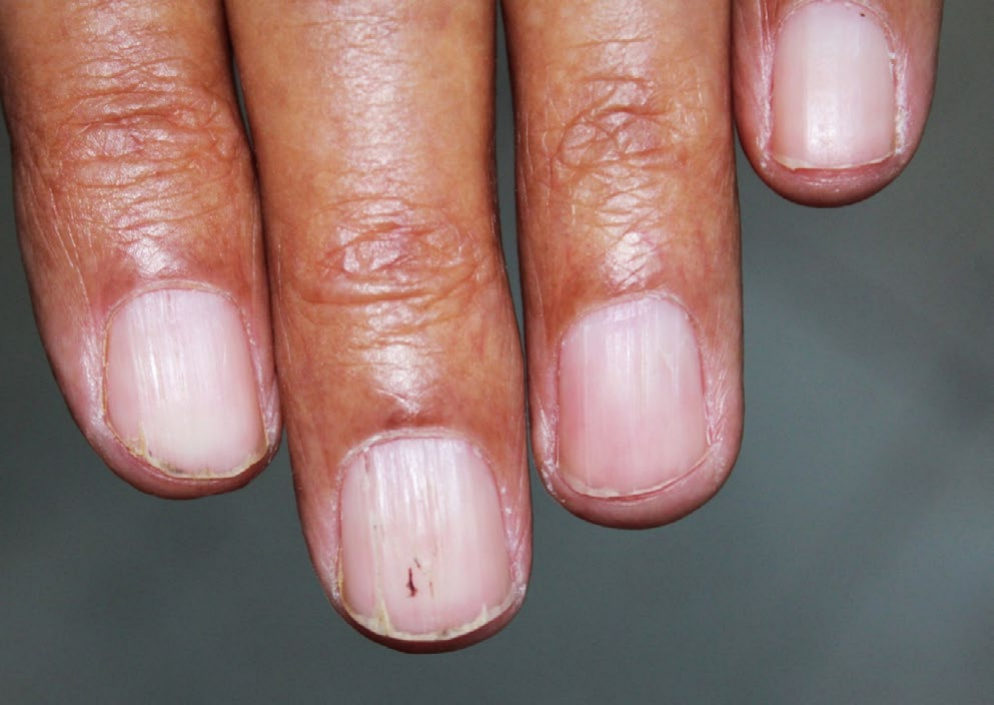
Physical examination revealed uniformly thinned and longitudinally ridged fingernails

**FIGURE 2:**
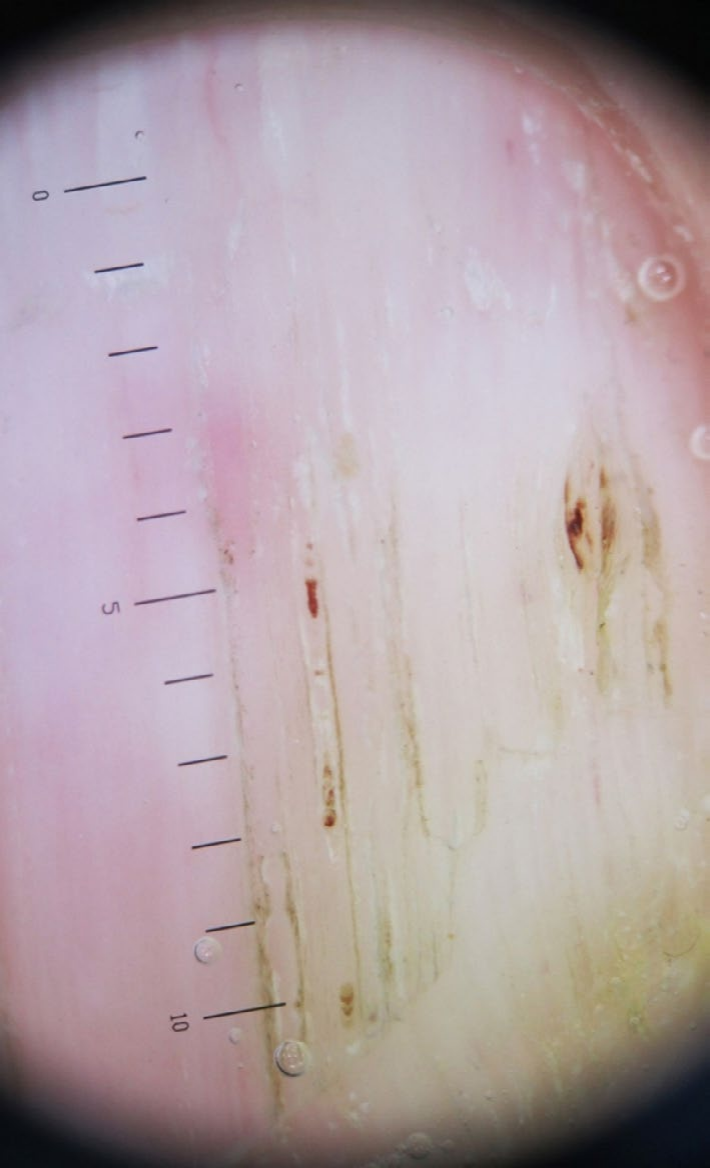
Dermoscopic images revealing longitudinally ridged fingernails and splinter hemorrhage

**FIGURE 3:**
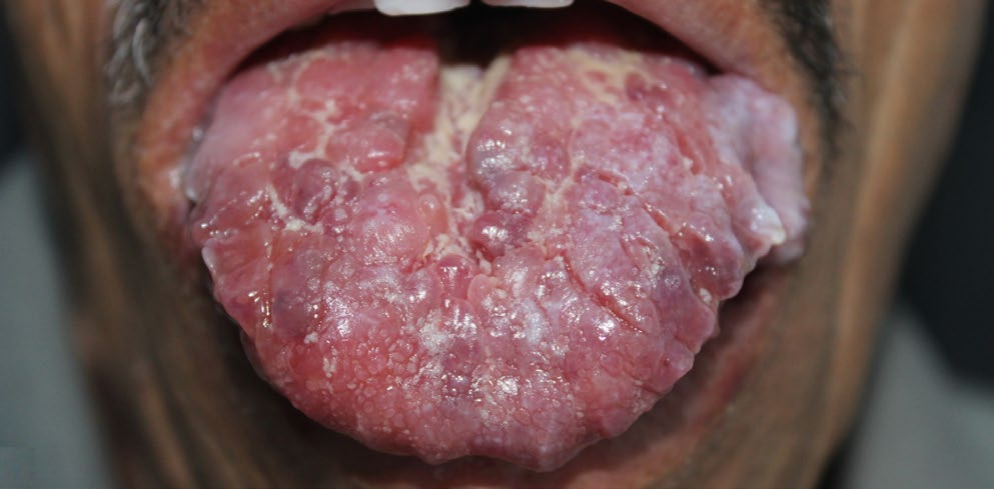
Macroglossia associated with hemorrhagic translucent papulo‐nodules

A 76‐year‐old patient presented with an 8‐month history of nail changes. He reported otherwise a history of weight loss evolving for a year. Dermatologic examination revealed uniformly thinned and longitudinally ridged fingernails (Figure 1). On close observation, splinter hemorrhages were found and were better identified using dermoscopy (Figure 2). The rest of dermatological examination revealed periorbital purpura. The tongue was uniformly enlarged and firm with hemorrhagic translucent papules (Figure 3). Physical examination revealed liver enlargement and edema of both lower limbs. Laboratory tests showed proteinuria and hypoalbuminemia. Serum protein immunoelectrophoresis showed a monoclonal gammopathy. Bone marrow examination revealed plasmacytic infiltration exceeding 20%. Skin biopsy of periorbital purpura showed extensive amyloidal deposits within the dermis. Diagnosis of systemic amyloidosis associated with multiple myeloma, with kidney and soft tissue involvement was made. The patient was referred to the hematology department for treatment.

Systemic amyloidosis results from the abnormal extracellular deposition of amyloid protein. Different types of systemic amyloidosis vary on the basis of the amyloidogenic precursor protein. The most common types are AL amyloidosis (amyloid light chains); AA amyloidosis (amyloid‐associated); and ATTR amyloidosis (transthyretin‐associated).^1^, ^2^ Systemic AL amyloidosis is associated with a wide spectrum of organ involvement and can present with a myriad of cutaneous manifestations.^1^ The most common of them are periorbital purpura, macroglossia and waxy, translucent or purpuric papules. Nail involvement is rarely described and can be the unique sign of the disease.^3^ It is usually characterized by brittleness, increased fragility, trachyonychia, longitudinal ridging, and subungual splinter hemorrhages.^3^, ^4^ Distal onycholysis and anonychia have also been described. The main differential diagnosis is lichen planus. Histologically, amyloid deposits are found in the dermis of the nail bed and matrix.^5^ In conclusion, trachyonychia with splinter hemorrhages in elderly should lead to considering the diagnosis of systemic amyloidosis.

## CONFLICTS OF INTEREST

None declared.

## AUTHOR CONTRIBUTION

NL wrote the first draft of the manuscript and took clinical pictures. IC helped in writing the manuscript, literature search, and corresponding author. MS helped in writing the manuscript and literature search. MSH and FZ revised and approved the final version of the manuscript. All the authors contributed to and have approved the final manuscript.

## Data Availability

Data sharing not applicable to this article as no datasets were generated or analyzed during the current study.
